# *Plasmodium falciparum* Reticulocyte Binding-Like Homologue Protein 2 (PfRH2) Is a Key Adhesive Molecule Involved in Erythrocyte Invasion

**DOI:** 10.1371/journal.pone.0017102

**Published:** 2011-02-28

**Authors:** Tajali Sahar, K. Sony Reddy, Mitasha Bharadwaj, Alok K. Pandey, Shailja Singh, Chetan E. Chitnis, Deepak Gaur

**Affiliations:** Malaria Group, International Centre for Genetic Engineering and Biotechnology (ICGEB), New Delhi, India; Institut National de la Santé et de la Recherche Médicale - Institut Cochin, France

## Abstract

Erythrocyte invasion by *Plasmodium* merozoites is a complex, multistep process that is mediated by a number of parasite ligand-erythrocyte receptor interactions. One such family of parasite ligands includes the *P. falciparum* reticulocyte binding homologue (PfRH) proteins that are homologous with the *P. vivax* reticulocyte binding proteins and have been shown to play a role in erythrocyte invasion. There are five functional PfRH proteins of which only PfRH2a/2b have not yet been demonstrated to bind erythrocytes. In this study, we demonstrated that native PfRH2a/2b is processed near the N-terminus yielding fragments of 220 kDa and 80 kDa that exhibit differential erythrocyte binding specificities. The erythrocyte binding specificity of the 220 kDa processed fragment of native PfRH2a/2b was sialic acid-independent, trypsin resistant and chymotrypsin sensitive. This specific binding phenotype is consistent with previous studies that disrupted the PfRH2a/2b genes and demonstrated that PfRH2b is involved in a sialic acid independent, trypsin resistant, chymotrypsin sensitive invasion pathway. Interestingly, we found that the smaller 80 kDa PfRH2a/2b fragment is processed from the larger 220 kDa fragment and binds erythrocytes in a sialic acid dependent, trypsin resistant and chymotrypsin sensitive manner. Thus, the two processed fragments of PfRH2a/2b differed with respect to their dependence on sialic acids for erythrocyte binding. Further, we mapped the erythrocyte binding domain of PfRH2a/2b to a conserved 40 kDa N-terminal region (rPfRH2_40_) in the ectodomain that is common to both PfRH2a and PfRH2b. We demonstrated that recombinant rPfRH2_40_ bound human erythrocytes with the same specificity as the native 220 kDa processed protein. Moreover, antibodies generated against rPfRH2_40_ blocked erythrocyte invasion by *P. falciparum* through a sialic acid independent pathway. PfRH2a/2b thus plays a key role in erythrocyte invasion and its conserved receptor-binding domain deserves attention as a promising candidate for inclusion in a blood-stage malaria vaccine.

## Introduction

Malaria is one of the major killer diseases of the world, which causes an annual mortality of around one million deaths primarily in infants and children less than 5 years [1]. The most severe and fatal form of malaria, cerebral malaria is caused by the apicomplexan parasite, *P. falciparum*. *P. vivax* is the other major *Plasmodium* species that causes human malaria. *P.vivax* primarily invades Duffy positive human erythrocytes, which is mediated by the interaction of the *P. vivax* Duffy binding protein (PvDBP) with the Duffy antigen (DARC) [2]–[4]. Unlike *P.vivax*, *P. falciparum* has developed a high level of redundancy in receptor usage, which enables erythrocyte invasion through multiple pathways [5]–[7].

Among the many parasite molecules that mediate erythrocyte invasion by *P*. *falciparum*, there are two major protein families, namely the Duffy binding-like family (DBL) and the Reticulocyte binding homologues (RH) that have been shown to bind erythrocytes [5]–[7]. The DBL family consists of five *P. falciparum* homologues of PvDBP, namely, EBA-175, EBA-140 (BAEBL), EBA-181 (JESEBL), EBL-1 and EBA-165 (PEBL) [5]–[7]. The PfRH family consists of six *P. falciparum* homologues of the *P. vivax* reticulocyte binding proteins (PvRBP), namely, PfRH1, PfRH2a, PfRH2b, PfRH3, PfRH4 and PfRH5 [5]–[7]. The DBL proteins are characterized by the presence of a conserved cysteine-rich region that mediates erythrocyte binding [4], [8]. While the DBLs have been studied extensively, the PfRH proteins have been discovered relatively recently and their functional characteristics have yet to be completely defined.

PfRH2a and PfRH2b [9], [10] were the first members of the PfRH family to be identified followed by PfRH1 [11], PfRH4 [12] and PfRH5 [13]. Like EBA-165 [14], PfRH3 is also a pseudogene that is transcribed but not translated [15]. PfRH2a and PfRH2b are encoded by two genes that due to their high sequence identity appear to have arisen from a duplication event. Both genes are located adjacently on chromosome 13 in a head to head orientation [9], [10], [16], [17]. These two genes are around 9 kb in length and share an identical 8 kb region which codes for 2700 amino acids. The difference in the gene sequences arises towards the 3′end that accounts for around 500 amino acids towards the C-terminal end. This unique region comprises of a part of the ectodomain, the transmembrane region and the cytoplasmic tail [9], [10], [16], [17]. The identical ectodomain of PfRH2a and PfRH2b comprising of 2700 amino acids is referred here as PfRH2a/b.

The interest in the PfRH family of proteins has been generated by recent work that has shown that the expression of these proteins correlates with the invasion phenotypes of different parasite lines [16], [18]–[21]. This was clearly demonstrated for PfRH4, whose upregulation was found to mediate a switch from a sialic acid dependent to sialic acid independent invasion phenotype in case of the *P. falciparum* clone, Dd2 [19], [20]. Similarly, it has been reported that PfRH proteins are differentially expressed between parasite lines with different invasion phenotypes [16], [18], [21]. The sialic acid dependent lines such as Dd2, MCamp and FCR3 express higher levels of PfRH1 and low levels of PfRH2a/2b [16], [18], [21]. On the other hand, the sialic acid independent lines such as 3D7, HB3, 7G8 express higher levels of PfRH2a/2b and lower levels of PfRH1 [16], [18], [21]. PfRH1, PfRH4 and PfRH5 have been shown to exhibit erythrocyte binding activity [11], [13], [22]–[24]. The erythrocyte binding domains of PfRH4 [23], [24] and PfRH1[22] have also been elucidated. Thus, among the five functional PfRH proteins, all except PfRH2a/2b have been shown to bind erythrocytes.

While previously the erythrocyte binding activity of PfRH2a/2b could not be detected [10], [16], the genetic disruption of PfRH2a and PfRH2b demonstrated a role for PfRH2a/b in erythrocyte invasion [16]. A comparison of the invasion phenotypes of the PfRH2b knockout and wild type parasite lines showed that PfRH2b mediates erythrocyte invasion through a sialic acid independent, trypsin resistant, chymotrypsin sensitive pathway [16]. In the same study, the disruption of the PfRH2a gene did not produce any differences in invasion phenotypes between the knockout and wild type parasites, implying that PfRH2a does not play a primary role in this invasion pathway [16]. Further, a recent study has reported that PfRH2a is also involved in sialic acid independent invasion although PfRH2b has a more critical role in this invasion pathway [25].

Based on this data, it appears that the PfRH2a and PfRH2b proteins could also possess erythrocyte binding activity mediated through regions in their ectodomains like other members of the PfRH family. In this study, we report that full length native PfRH2a/2b is processed in two steps that yield smaller fragments. Further, we demonstrate for the first time that different processed fragments of native PfRH2a/b bind erythrocytes with different specificities. In addition, we have identified a 40 kDa N-terminal region (rPfRH2_40_), common to both PfRH2a and PfRH2b, which binds erythrocytes with the same specificity as the native parasite protein. This region of the PfRH2a/b native protein appears to constitute its receptor binding domain. Our study is the first to report the erythrocyte binding activity of PfRH2a/b and delineate a region of the native protein that mediates its binding with erythrocytes. We also demonstrate that the rPfRH2_40_ domain is immunogenic. The antibodies raised against this functional domain recognize the native parasite protein and inhibit erythrocyte invasion by *P. falciparum in vitro.* The ability of antibodies raised against the functional domain of PfRH2a/b to inhibit erythrocyte invasion suggests that it may be useful to include rPfRH2_40_ in a blood-stage vaccine against *P. falciparum* malaria.

## Materials and Methods

### Ethics Statement

The animal studies described below were approved by the ICGEB Institutional Animal Ethics Committee (IAEC Reference No. MAL-51). ICGEB is licensed to conduct animal studies for research purposes under the registration number 18/1999/CPCSEA (dated 10/1/99).

### Cloning of the PfRH2 constructs

A codon optimized gene of 1098 bp encoding 366 amino acids (Ser 495 to Ile 860) of PfRH2a/b from *P. falciparum* 3D7 (rPfRH2_40_) with a C-terminal 6-histidine (6-His) tag was synthesized. The regions of the native PfRH2a/b gene encoding the amino acid sequence of rRH2-Pro1 (Met 76 to Asp 494) and rRH2-Pro4 (Asn 1599 to Ile 2059) were PCR amplified from 3D7 genomic DNA using the following primers: RH2-Pro1F: GTGTgctagcAAGAGATCG CTTATAAATTTAG, RH2-Pro1R: TCTCctcgagATCCAG ATTTGCTTTTGGAT; RH2-Pro4F: GTGTgctagcAACATTAAAAGAGAAGGTGATG, RH2-Pro4R: TCTCctcgagAATATTATTTG AAATATCATTTTCTCT. The synthetic gene encoding rPfRH2_40_ and the PCR products encoding rRH2-Pro1 and rRH2-Pro4 were digested with *Nhe* I and *Xho* I (New England Biolabs, Beverly, MA) and inserted downstream of the T7 promoter in the *E. coli* expression vector, pET-24b (Novagen, San Diego, CA) to obtain the plasmids pPfRH2_40_-pET24b, pPfRH2-Pro1-pET24b, pPfRH2-Pro4-pET24b respectively. Sequencing of the ligated plasmids confirmed the correct sequence of the *PfRh2a/b* gene fragments and that the insertions were in the correct reading frame.

### Expression and purification of recombinant proteins and generation of specific antisera

*E. coli* BL21(DE3) cells (Novagen, San Diego, CA) were transformed with the expression plasmids and used to produce the recombinant PfRH2a/b proteins. Transformed *E. coli* BL21(DE3) were cultured in Luria broth at 37°C. Expression of rPfRH2_40_, rRH2-Pro1 and rRH2-Pro4 proteins was induced with 1 mM IPTG when OD_600_ of the culture was in the range of 0.6 to 0.8. Cells were grown for 4 hours after induction and were harvested by centrifugation at 3000 g. Harvested cell pellets were lysed by sonication and each of the three overexpressed proteins were found in inclusion bodies. The inclusion bodies were collected by centrifugation at 15000 g and solubilized in 8 M Guanidine-HCL. The proteins were purified from solubilized inclusion bodies by Ni-NTA (nitrilotriacetic acid) affinity chromatography using standard methods and further purified as described below.

Metal-affinity purified rPfRH2_40_ protein was refolded by a 30-fold rapid dilution in an L-Arginine based refolding buffer. The refolding solution was incubated for 24 hours at 4°C with continuous stirring, and then dialyzed against phosphate buffered saline (PBS, pH 7.4). The dialyzed protein solution was applied on a Q-sepharose anion exchange column (GE Healthcare, Piscataway, NJ) to purify rPfRH2_40_.

Metal affinity purified (Ni-NTA) PfRH2a/b proteins – rRH2-Pro1 and rRH2-Pro4 were run in an SDS-PAGE gel and the proteins were eluted from the acrylamide for injection into mice. The immunization schedule for these two proteins was the same as followed for rPfRH2_40_. Mice and rabbits were immunized intramuscularly with 25 µg and 100 µg respectively of the recombinant proteins emulsified with complete Freund's adjuvant (Sigma, St. Louis, MO) on day 0 followed by two boosts emulsified with incomplete Freund's adjuvant on days 28 and 56. The sera were collected on day 70. Antibody levels were measured in the sera by ELISA [26].

### Erythrocyte Binding Assays (EBA)

Erythrocyte binding assays were performed as described earlier [23]. Soluble parasite proteins were obtained from *P. falciparum* 3D7 culture supernatants of schizont-infected erythrocytes as described previously [23]. Briefly, culture supernatants or recombinant proteins were incubated with human erythrocytes at 37°C following which the suspension was centrifuged through dibutyl phthalate (Sigma, St. Louis, MO

The supernatant and oil were removed by aspiration. Bound parasite proteins were eluted from the erythrocytes with 1.5 M NaCl. The eluate fractions were analyzed for the presence of the PfRH2a/b protein by immunoblotting using anti-rPfRH2_40_ or anti-rRH2-Pro1 antibodies.

### Growth inhibition assays (GIA)

In the GIA assays, 6×10^4^ schizont-infected erythrocytes were incubated with 2×10^7^ target erythrocytes in 100 µl of complete RPMI medium containing IgG (5 mg/ml) purified from anti-rPfRH2_40_ rabbit sera. The efficacy of the antibodies was compared with control IgG purified from rabbits immunized with an unrelated peptide formulated with the same Freund's adjuvant. Rabbit IgG was purified on protein G sepharose (GE Healthcare, Uppsala, Sweden) and dialyzed against RPMI. Parasitemia in cultures was estimated after an incubation of 40 hours (one cycle of invasion in target erythrocytes) by flow cytometry as described earlier [23], [24]. Parasitemia observed in presence of anti-rPfRH2_40_ IgG and control IgGs (5 mg/ml) were used to determine invasion inhibition rates as described previously [23], [24].

## Results

### Expression and refolding of a recombinant 40 kDa protein fragment derived from PfRH2a/b (rPfRH2_40_) and generation of specific PfRH2a/b antibodies

**Figure 1:**
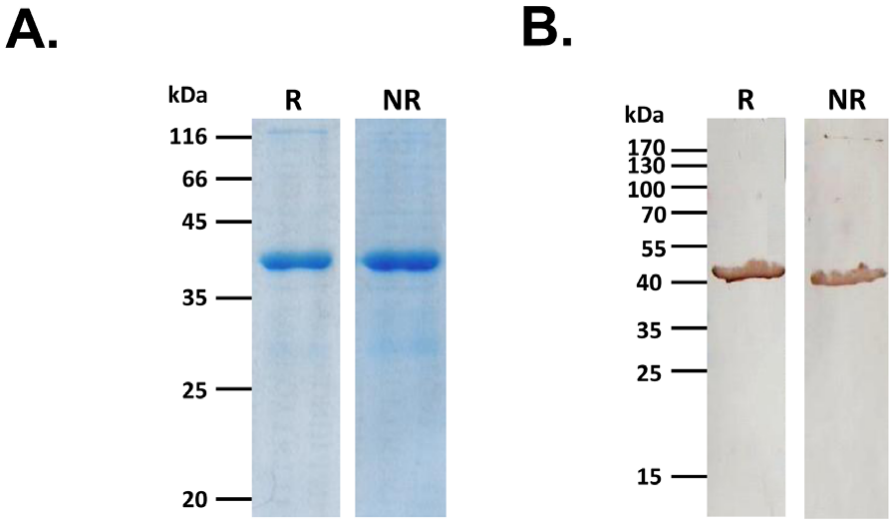
Expression and purification of the recombinant rPfRH2_40_ protein. The 366 amino acid region (Ser495 to Ile860) common to both PfRH2a and PfRH2b was expressed and purified to homogeneity. (A) The purified protein was analyzed on an SDS-PAGE under reducing (R) and non-reducing (NR) conditions. (B) The purified recombinant protein was detected in immunoblots under reducing and non-reducing conditions by using an anti-His tag antibody.

**Figure 2:**
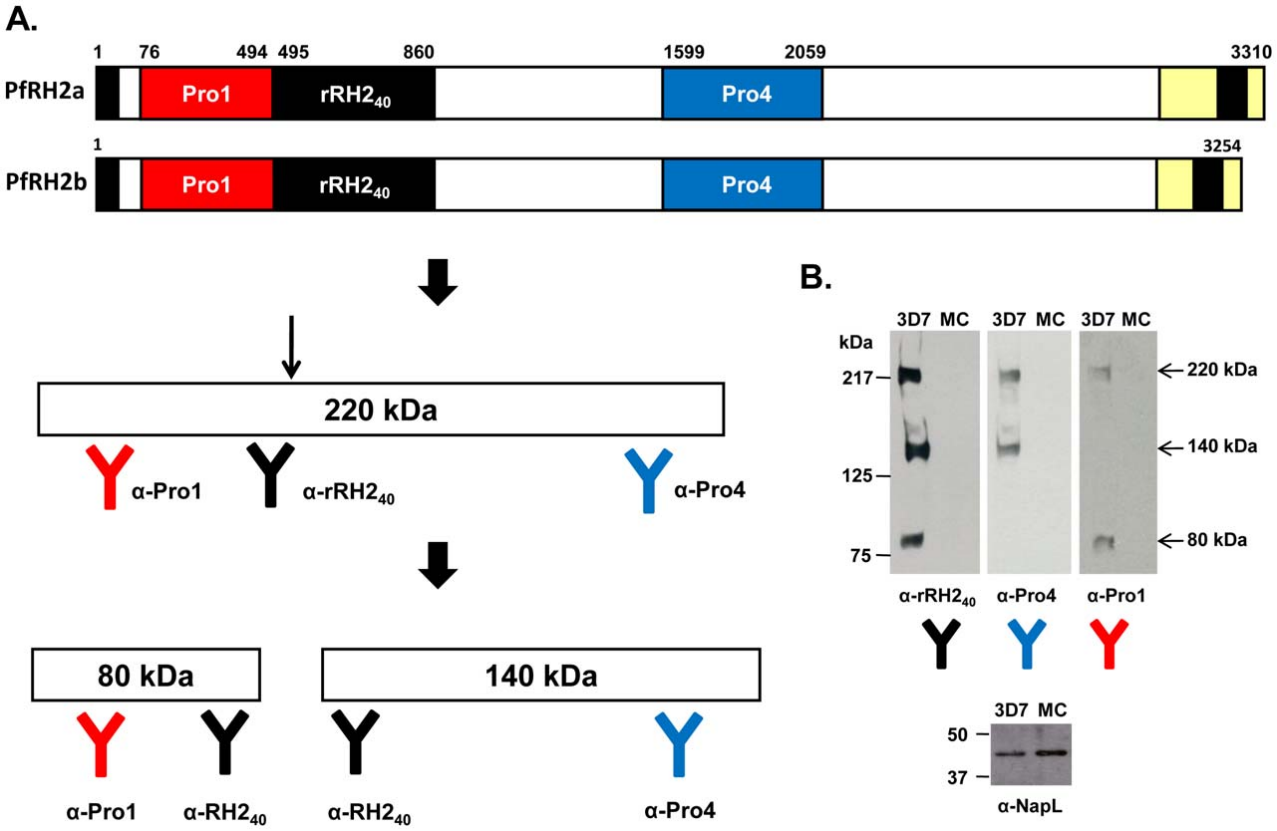
The native PfRH2a/b protein is processed and its expression varies between different parasite lines. (A) A schematic representation of the protein structure of PfRH2a/2b and the different regions against which specific PfRH2a/b antibodies have been raised are shown. In line with the immunoblot results, the steps of processing and the resulting PfRH2a/b fragments are depicted. Native full length PfRH2a/b undergoes processing to produce a 220 kDa fragment, which is further processed between the amino acids 495–860 to produce two fragments of 80 kDa and 140 kDa. (B) Immunoblots of parasite extracts from the 3D7 and MCamp clones detected by the three PfRH2a/b antibodies. The three antibodies detected PfRH2a/b processed fragments (marked by arrows) only in 3D7, a sialic acid independent clone, and not in MCamp, a sialic acid dependent clone. Antiserum against NapL, a nuclear accessory protein, was used to confirm that an equal amount of parasite extract was used in the immunoblots.

A 366 amino acid sequence (Ser 495 to Ile 860) of PfRH2a/b (∼40 kDa) was chosen for expression on the basis of separate pair wise clustal alignments of PfRH2b (Genbank AAN39447) with PfRH1 (Genbank AAQ10315) (Figure S1) and PfRH4 (Genbank AAM47174) (Figure S2). The pair wise alignments showed regions of homology in PfRH2a/2b with the erythrocyte binding domains of PfRH1 and PfRH4 (Figures S1 and S2). The homologous region is common to both PfRH2a and PfRH2b and lies within the 2700 amino acid region of their ectodomains that is identical (PfRH2a/b). The recombinant 40 kDa fragment of PfRH2a/b (rPfRH2_40_) was expressed with a C-terminal 6-His tag in *E. coli* in inclusion bodies, refolded after purification under denaturing conditions by metal affinity chromatography and further purified to homogeneity using ion-exchange chromatography (Figure 1A). Recombinant rPfRH2_40_ was identified in immunoblots using an anti-His-tag specific antibody (Figure 1B) confirming expression of the full length 366 amino acid recombinant rPfRH2_40_ protein. Mice and rabbits were immunized with the recombinant rPfRH2_40_ protein to produce PfRH2a/b-specific antibodies. High titer antibodies against the recombinant rPfRH2_40_ protein were detected in both mice and rabbits with end points observed at dilutions of 1∶320,000 and 1∶640,000, respectively (Figure S3). In addition, we also raised PfRH2a/b mice antibodies against other regions of the protein, rRH2-Pro1 (amino acids 76–494) and rRH2-Pro4 (amino acids 1599–2059) (Figures 2A and S4). As the protein sequence of rPfRH2_40_, rRH2-Pro1 and rRH2-Pro4 is completely identical between the two PfRH2 homologues, our antibodies cannot distinguish between PfRH2a and PfRH2b. Thus, we have termed the parasite protein detected by these antibodies as PfRH2a/b as they detect the common region from both proteins, although PfRH2b has been demonstrated to play a more dominant role in invasion than PfRH2a [16], [25].

The specificity of the anti-rPfRH2_40_ antibodies was checked by localization studies of the PfRH2a/b native protein in merozoites by immunofluorescence confocal microscopy (details are given in Materials and Methods S1) using the anti-rPfRH2_40_ polyclonal mouse sera (Figure S5). The native PfRH2a/b was found to be localized at the apical pole of the merozoite (Figure S5). PfRH2a/b co-localizes with the known rhoptry bulb marker protein, clag3.1 demonstrating that it is localized in the rhoptries (Figure S5A). On the other hand there was no co-localization with the micronemal marker EBA-175 (Figure S5B). Our data is consistent with previous reports that have localized PfRH2a/2b in the rhoptries by immunoelectron microscopy [16] and confirms the specificity of our PfRH2a/b antibodies.

### Detection of processed forms of native PfRH2a/b

To further confirm the specificity of our anti-PfRH2a/b antibodies, we performed immunoblots using detergent extracts made from late stage schizonts of *P. falciparum* lines, 3D7 and MCamp (Figure 2B). The anti-rPfRH2_40_ antibodies detected three native proteins by immunoblotting in the range of 220 kDa, 140 kDa and 80 kDa. These proteins were detected only in the 3D7 parasite clone but not in MCamp (Figure 2B). Detection of a PfRH2a/b protein fragment of around 220 kDa is consistent with previous reports [10], [16], [25]. Full length native PfRH2a/b is a 350 kDa protein [9], [10]. Presumably the smaller size reflects proteolytic cleavage of PfRH2a/b produced during schizont maturation and merozoite formation. To confirm the specificity of the three protein fragments, we performed immunoblotting with the anti-PfRH2a/b polyclonal antibodies raised against different regions of PfRH2a/b, termed as rRH2-Pro1 and rRH2-Pro4 (Figure 2A). rPfRH2_40_ antibodies were raised against amino acids 495 to 860; rRH2-Pro1 antibodies were raised against the region upstream of rPfRH2_40_ comprising of amino acids 76 to 494; rRH2-Pro4 antibodies were raised against a region downstream of rPfRH2_40_ comprising of amino acids 1599 to 2059 (Figure 2A). We observed that anti-rRH2Pro1 antibodies detected two protein fragments of 220 kDa and 80 kDa out of the three detected by the anti-rPfRH2_40_ antibodies (Figure 2B). On the other hand, antibodies raised against rRH2-Pro4 detected the 220 kDa and 140 kDa proteins only (Figure 2B). The 220 kDa fragment was detected by all three antibodies confirming its specificity. Apart from the rPfRH2_40_ antibodies, the lower fragments of 80 kDa and 140 kDa were detected only by the anti-rRH2Pro1 and anti-rRH2Pro4 antibodies, respectively (Figure 2B). The fact that the anti-rPfRH2_40_ antibodies detected all three fragments suggested that all three protein fragments share epitopes recognized by the polyclonal antibodies. These results implied that the 220 kDa native PfRH2a/b processed protein was being further processed in to the 80 kDa and 140 kDa fragments and that the processing was occurring in between the rPfRH2_40_ region (Figures 2A and B).

Proteolytic processing has been reported for a number of invasion related parasite proteins [11], [13], [22]–[24], [27]–[29]. We have now demonstrated that like its other PfRH homologues, the 350 kDa native PfRH2 also undergoes processing. Using a higher concentration of anti-rPfRH2_40_ mouse sera as well as higher amount of parasite extract from 3D7, we were able to detect bands in the range of 350 kDa which appear to represent the unprocessed native protein (Figure S6A). Detection of processed fragments of native PfRH2a/b in line with previous studies [10], [16], [25] further confirms the precise specificity of our anti-PfRH2a/b antibodies.

Further our data is consistent with previous studies that have shown higher expression of PfRH2a/2b in strains like 3D7 that use sialic acid independent invasion pathways compared to strains like MCamp that invade by sialic acid dependent pathways [16], [18], [21]. All the three PfRH2a/b antibodies detected native protein only in 3D7 and not in MCamp. As a control to confirm that equal amounts of parasite extracts were used in the immunoblots, we assayed the presence of a nucleosome assembly protein (NapL), which has a house keeping function and is expressed equally among different *P. falciparum* lines [30]. NapL was found to be present at similar levels in 3D7 and MCamp (Figure 2B), confirming that equal amounts of 3D7 and MCamp parasite extract were used. Thus, our results are in agreement with previous reports on the expression of PfRH2a/2b and confirm the specificity of our anti-PfRH2a/b sera.

### Native PfRH2a/b and recombinant rPfRH2_40_ exhibit erythrocyte binding activity

**Figure 3:**
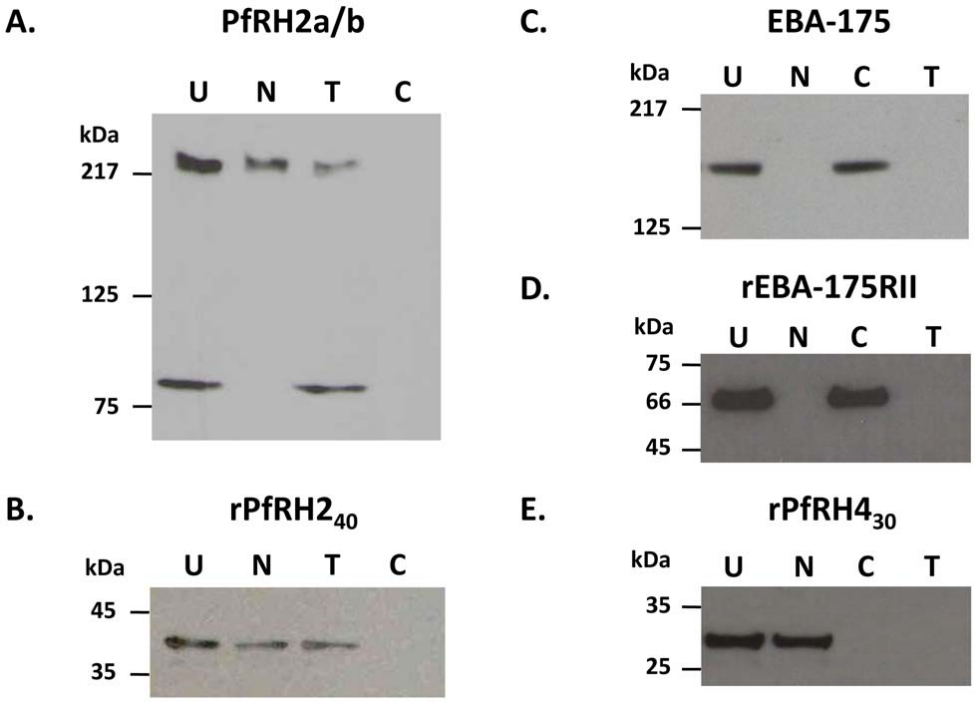
Erythrocyte binding activity of native PfRH2a/b and recombinant rPfRH2_40_ proteins. (A) Binding of the native PfRH2a/b protein in 3D7 culture supernatants incubated with untreated (U) erythrocytes, different enzyme-treated erythrocytes (Nm: neuraminidase-treated; T: trypsin-treated; C: chymotrypsin-treated). The processed PfRH2a/b parasite protein fragments (220 kDa and 80 kDa) were detected in the eluate fractions by immunoblotting using the anti-rPfRH2_40_ antibodies. (B) Binding of the recombinant rPfRH2_40_ protein with a similar set of erythrocytes. (C) Binding of native EBA-175 from 3D7 parasite culture supernatant with different enzyme treated erythrocytes. (D) Binding of recombinant EBA-175 region II (rEBA-175 RII) with enzyme treated erythrocytes. (E) Binding of recombinant PfRH4 (rRH4_30_) region (amino acids 328–588) with enzyme treated erythrocytes.

The erythrocyte binding characteristics of native PfRH2a/b was studied in standard erythrocyte binding assays using different enzyme treated erythrocytes and culture supernatants of purified *P. falciparum* schizonts [31], [32]. The processed fragments of PfRH2a/b observed in the parasite detergent based extract were also found in the culture supernatants. The presence of native PfRH2a/b among the proteins eluted from the human erythrocytes incubated with parasite culture supernatants was detected by immunoblotting using anti-PfRH2a/b antibodies (Figures 3A and S6B) and interestingly exhibited differential erythrocyte binding activity.

The native 220 kDa and 80 kDa processed fragments of PfRH2a/b were observed to exhibit differential erythrocyte binding activity. The native 220kDa PfRH2a/b fragment from culture supernatants bound normal human erythrocytes as well as neuraminidase-treated erythrocytes and trypsin treated erythrocytes (Figures 3A and S6B). The binding of the 220 kDa PfRH2a/b fragment to erythrocytes was abolished by chymotrypsin treatment implying that its erythrocyte receptor was chymotrypsin sensitive (Figures 3A and S6B). Thus, the erythrocyte binding specificity of the 220 kDa larger fragment of PfRH2a/b was sialic acid independent, trypsin resistant and chymotrypsin sensitive. On the other hand, in the same binding experiment, we observed that the smaller 80 kDa PfRH2a/b fragment exhibited a different erythrocyte binding phenotype (Figures 3A and S6B). The 80 kDa fragment bound only with untreated erythrocytes and trypsin treated erythrocytes but failed to bind with neuraminidase treated and chymotrypsin treated erythrocytes (Figures 3A and S6B). Thus the binding phenotype of the 80 kDa smaller processed fragment of PfRH2a/b was sialic acid dependent, trypsin resistant and chymotrypsin sensitive.

In parallel control experiments with the same eluate samples, native EBA-175 bound untreated erythrocytes and chymotrypsin treated erythrocytes, but not neuraminidase treated erythrocytes and trypsin treated erythrocytes (Figure 3C). The EBA-175 parasite protein is known not to bind neuraminidase treated erythrocytes or trypsin treated erythrocytes as its receptor, glycophorin A, is cleaved after these enzymatic treatments [8]. This result confirms that non-specific binding of parasite culture supernatant with the erythrocytes was not observed in our assay. Thus, native PfRH2a/b appears to bind human erythrocytes with a differential specificity with the different processed fragments of PfRH2a/b exhibiting a difference in their dependence on sialic acids to bind human erythrocytes. A number of previous reports have demonstrated PfRH2 to be involved in sialic acid independent invasion [16], [17], [25] and to be highly expressed in sialic acid independent clones compared to sialic acid dependent clones [16], [18], [21]. The erythrocyte binding phenotype of the 220 kDa processed fragment is consistent with these former reports. While in this context, the physiological significance of the erythrocyte binding profile of the 80 kDa fragment is yet not clear, it may be possible that the same protein after processing may be interacting with different erythrocyte receptors.

The erythrocyte binding activity of rPfRH2_40_ was also studied in similar erythrocyte binding assays. The rPfRH2_40_ recombinant protein also bound erythrocytes with the same specificity as the native 220 kDa PfRH2a/b protein (Figure 3B) in that it bound neuraminidase treated and trypsin treated erythrocytes but not chymotrypsin treated erythrocytes. Along with rPfRH2_40_, we also examined the erythrocyte binding of two recombinant proteins – EBA-175 Region II (rEBA-175RII) and PfRH4 (rPfRH4_30_) as controls in the same assay [23], [33]. EBA175 RII is the erythrocyte binding domain of the native EBA-175 protein [8]. Thus, similar to the native protein, rEBA175RII was observed to bind untreated erythrocytes and chymotrypsin treated erythrocytes, but not to neuraminidase treated erythrocytes and trypsin treated erythrocytes (Figure 3D). The PfRH4 recombinant protein, rPfRH4_30_, has been reported earlier to bind erythrocytes in sialic acid independent (neuraminidase resistant), trypsin and chymotrypsin sensitive manner [23], [24]. Consistent with previous reports, we found that the recombinant rPfRH4_30_ protein bound neuraminidase treated erythrocytes but failed to bind with trypsin or chymotrypsin treated erythrocytes (Figure 3E). These control binding assays demonstrated the erythrocyte binding characteristics of known invasion proteins consistent with their previously reported phenotypes confirming the specificity of our erythrocyte binding assay and ensuring the optimal enzymatic treatment of the erythrocytes.

### Antibodies to rPfRH2_40_ blocked erythrocyte invasion

**Figure 4:**
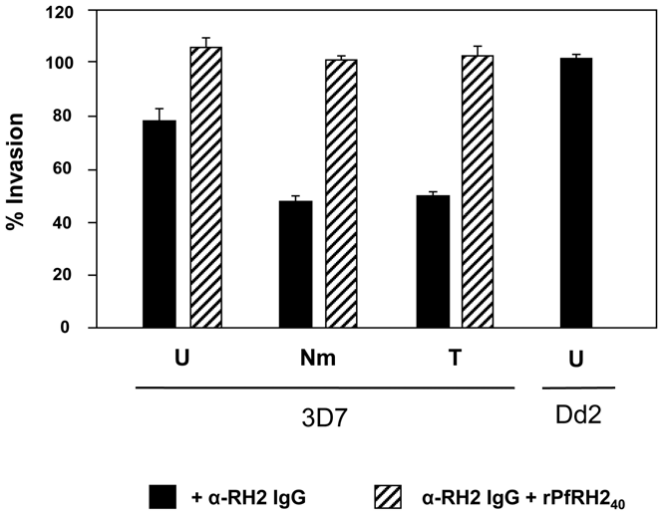
Invasion inhibitory activity of anti-rPfRH2_40_ antibodies. Rabbit purified IgG against rPfRH2_40_ was tested for its invasion inhibitory activity against the 3D7 and Dd2 parasite clones. Rabbit anti-PfRH2_40_ IgG blocked invasion of normal untreated erythrocytes by the 3D7 parasite line by 25% and with neuraminidase treated erythrocytes and trypsin treated erythrocytes, the percent inhibition was 50–53%. The anti-rPfRH2_40_ antibodies did not exhibit any invasion inhibition effect on Dd2 with untreated erythrocytes. The invasion inhibitory activity with neuraminidase treated erythrocytes was not tested for Dd2 as the Dd2 parasite clone is sialic acid dependent and fails to invade neuraminidase treated erythrocytes. For studying the reversal of antibody inhibition, rPfRH2_40_, was added at a final concentration of 30 µg/ml.

Since PfRH2a/b is involved in erythrocyte invasion with an erythrocyte binding role and the rPfRH2_40_ region binds with the same specificity as the native protein, we tested if antibodies against rPfRH2_40_ could block erythrocyte invasion. We measured the invasion inhibitory activity of purified rabbit IgG raised against rPfRH2_40_ in standard GIA (growth inhibition assays) as described earlier [23], [24]. Anti-rPfRH2_40_ rabbit IgG blocked invasion of normal untreated erythrocytes by the 3D7 parasite line by 25% (Figure 4). Since, PfRH2a/b mediates invasion through the sialic acid independent, trypsin resistant pathway, we tested the GIA activity of the PfRH2a/b antibodies for invasion into neuraminidase and trypsin treated erythrocytes. The 3D7 parasite line has a sialic acid independent, trypsin resistant invasion phenotype. With neuraminidase treated erythrocytes and trypsin treated erythrocytes, the percent inhibition (Figure 4) was higher (50–53%) than that observed for invasion into normal erythrocytes indicating that under conditions where the 3D7 parasite is restricted to invade through only sialic acid independent or trypsin resistant pathways, the invasion inhibition was more efficient. Further, the invasion inhibition of the purified IgG could be neutralized by the addition of recombinant rPfRH2_40_ protein (30 µg/ml), which clearly demonstrated that the invasion inhibition was specifically due to the anti- rPfRH2_40_ antibodies. To further confirm the GIA specificity of our antibodies, we tested the GIA activity of the anti-PfRH2_40_ antibodies with the *P. falciparum* clone Dd2 that invades only through sialic acid dependent pathways (Figure 4). The anti-rPfRH2_40_ antibodies did not exhibit any invasion inhibition effect on Dd2 (Figure 4), which is consistent with the fact that PfRH2 is not involved in sialic acid dependent invasion pathways and further confirms the specific invasion inhibitory effect of anti-PfRH2a/b antibodies.

## Discussion

*P. falciparum* exhibits a redundancy in its repertoire of invasion molecules that enables it to invade human erythrocytes through multiple pathways [5]–[7]. One family of parasite molecules that mediates *P. falciparum* erythrocyte invasion is the *P. falciparum* reticulocyte homology (PfRH) proteins [5]–[7]. These proteins are homologous to the *P. vivax* reticulocyte binding proteins (PvRBP-1/2) which mediate specific invasion of reticulocytes by *P. vivax* [34]. *P. falciparum* does not exhibit any preference for reticulocyte invasion and the PfRH homologues bind mature human erythrocytes during invasion [5]–[7].

PfRH1 has been shown to bind erythrocytes in a sialic acid dependent, trypsin resistant, chymotrypsin resistant manner [11], [21], [22]. Like EBA-175, PfRH1 has been shown to be a major ligand for sialic acid dependent invasion as all sialic acid dependent clones express high levels of PfRH1 [21]. PfRH4 is another PfRH protein which has clearly been shown to bind erythrocytes [23], [24]. The importance of PfRH4 was realized by its upregulation in experiments that mediated an invasion switch in the Dd2 (W2mef) clone from sialic acid dependent to sialic acid independent pathways [19], [20]. PfRH4 has been shown to bind in a sialic acid independent, trypsin sensitive and chymotrypsin sensitive manner [23],[24]. PfRH5 is the third protein of the family that has been shown to bind human erythrocytes [13], [30]. PfRH5 has also been proven to mediate invasion of *Aotus* erythrocytes by *P. falciparum* [13]. Like EBA-140 [35] and EBA-181 [36], polymorphisms in PfRH5 have been shown to determine receptor binding specificity [13]. PfRH3 is a pseudogene that is transcribed but not translated [15].

Recently a number of reports have elucidated the erythrocyte binding region of PfRH proteins. The first receptor binding region of PfRH proteins was identified for PfRH4 [23]. This was followed by further studies on PfRH1 [22] and PfRH4 [24], which mapped the receptor binding region by analyzing the binding activity of several overlapping fragments of the PfRH proteins. PfRH1 and PfRH4 are homologous in their N-terminal regions, which have been shown to mediate erythrocyte binding [22]–[24].

Despite being a member of a family of erythrocyte binding proteins, PfRH2a/2b had not been shown to bind erythrocytes. While it was clear from previous reports that PfRH2a/2b has a role in invasion [16], this is the first report to demonstrate that PfRH2a/2b exhibits erythrocyte binding activity. We expressed and purified a 366 amino acid region near the N-terminal of the PfRH2a/b ectodomain (rPfRH2_40_). This region was chosen on the basis of its homology in pair wise clustal alignments with the erythrocyte binding regions of PfRH1 and PfRH4. Like the cysteine rich regions of the DBLs, PfRH proteins lack any highly conserved regions that would obviously appear to constitute a globular functional domain. The rPfRH2_40_ region contains one cysteine. The rPfRH2_40_ sequence of interest was codon optimized to obtain maximum expression in *E. coli*. The recombinant protein was expressed in an insoluble form as inclusion bodies and was refolded. The refolded and purified protein was used to immunize mice and rabbits to generate specific antibodies against PfRH2a/b. The recombinant protein was found to be highly immunogenic in both mice and rabbits.

Antibodies raised against rPfRH2_40_ localized the native proteins in the rhoptries. This is consistent with previous immune electron microscopy studies that demonstrated localization of PfRH2a/b in the rhoptries [16]. Further, in line with previous studies, we observed higher levels of expression of PfRH2a/b in a sialic acid independent clone 3D7 compared to a sialic acid dependent clone, MCamp. These results confirmed the specificity of our PfRH2a/b antibodies.

The anti-rPfRH2_40_ antibodies detected processed fragments of native PfRH2a/b protein in parasite extracts and culture supernatants by immunoblotting consistent with previous studies [10], [16], [25]. Proteolytic processing is a phenomenon that has been studied and well established for a number of parasite proteins involved in erythrocyte invasion such as MSP-1, AMA-1, PfRH1, PfRH4 and PfRH5 [11], [13], [21]–[24], [27]–[29], [37], [38]. Proteolytic processing of invasion ligands is mediated by a vast repertoire of parasite proteases such as rhomboids and subtilisins during invasion [37].

Polyclonal antibodies raised against rPfRH2_40_ (Ser 495 to Ile 860) detected protein fragments of around 220 kDa, 140 kDa and 80 kDa. The fact that anti-rPfRH2_40_ polyclonal antibodies specifically detected several fragments implied that all the three fragments contain epitopes that could be recognized by the antibodies. It thus appeared that the 220 kDa processed fragment of PfRH2a/b protein was being further processed into smaller fragments. This was confirmed by immunoblotting experiments using antibodies raised against different regions of PfRH2a/b. We raised polyclonal antibodies against two different regions of PfRH2, anti-rRH2Pro1 raised against amino acids 76–494 and anti-rRH2Pro4 raised against amino acids 1599–2059. The immunoblots of parasite extracts with these two antibodies confirmed our model of PfRH2 processing as shown in Figure 2A. All the three antibodies detected the 220 kDa processed fragment of native PfRH2. Further, antibodies only against rRH2-Pro1 (76–494) and rPfRH2_40_ (495–860) detected the 80 kDa fragment while the antibodies only against rPfRH2_40_ (495–860) and rRH2-Pro4 (1599–2059) detected the 140 kDa fragment. This clearly demonstrated that the 220 kDa native PfRH2a/b protein was being further processed between the amino acids 495–860 yielding the 80 kDa fragment towards the N-terminus and the 140 kDa fragment towards the C-terminus as shown in Figure 2A. While, we do not yet know the precise sites of processing of PfRH2a/b, we speculate that full length native PfRH2a/b might be processed between the amino acids 30–123 at the N-terminus and 2030–2123 towards the C-terminus to yield the 220 kDa fragment, which is further processed approximately between the amino acids 760–850 to yield the 80 kDa and 140 kDa fragments. The sequential processing observed for PfRH2 has been previously well reported for MSP-1 [27] and PfRH1 [38]. While our report is the first to demonstrate processing of PfRH2, more work will be required to understand the precise mechanism of PfRH2 processing and its physiological significance for the parasite.

While PfRH2 was observed to be processed, it was clear that only the 220 kDa and 80 kDa fragments exhibited erythrocyte binding activity. However the specificity of the binding was observed to be different with respect to their use of sialic acids as a component of their erythrocyte receptors. The erythrocyte binding specificity of the native 220 kDa PfRH2a/b fragment was sialic acid independent, trypsin resistant and chymotrypsin sensitive. This binding phenotype suggests that the erythrocyte receptor to which the 220 kDa PfRH2a/b protein binds does not comprise of sialic acid moieties as neuraminidase treatment had no effect on its erythrocyte binding. Similarly the receptor is trypsin resistant as the 220 kDa PfRH2a/b protein bound trypsin treated erythrocytes. However, since the binding of the 220 kDa PfRH2a/b protein was abolished with chymotrypsin treatment, its erythrocyte receptor is chymotrypsin sensitive. On the other hand, the smaller 80 kDa processed fragment of PfRH2a/b was observed to bind erythrocytes in a sialic acid dependent, trypsin resistant, chymotrypsin sensitive manner. The erythrocyte binding activity of both 220 kDa and the 80 kDa fragments was confirmed by PfRH2a/b antibodies raised against rPfRH2_40_ and rRH2-Pro1. Thus, while the differential erythrocyte binding activity of the two processed fragments of PfRH2 is a consistent observation, the physiological significance of this differential erythrocyte binding activity of the lower 80 kDa fragment is not clear. Our observations are similar to the case of erythrocyte binding of EBA-175 which has been shown to bind Glycophorin A in a sialic acid dependent manner [8], [31]. The full length protein was shown to bind erythrocytes in a culture supernatant based erythrocyte binding assay [31] and the domain (region II) responsible for mediating the binding has been identified and characterized [8]. However, in addition a 65 kDa processed fragment of EBA-175 has also been reported to bind erythrocytes in a sialic acid independent manner [39]. Thus while EBA-175 has been conclusively proven to bind glycophorin A and mediate invasion through the sialic acid dependent pathway, the physiological significance of the binding of the 65 kDa fragment remains unclear.

This specificity of erythrocyte binding of the native PfRH2a/b is consistent with a previous knockout study, which on the basis of differences in the invasion phenotypes between the PfRH2b knock out and wild type parasite lines, had demonstrated PfRH2b to play a role in a sialic acid independent, trypsin resistant, chymotrypsin sensitive invasion pathway [16]. Both PfRH2a and PfRH2b were knocked out individually in the 3D7 parasite clone and the invasion properties of the knock out parasites were assayed. The PfRH2b knock out line was observed to invade neuraminidase treated and trypsin treated erythrocytes at a lower efficiency suggesting that the loss of the gene had compromised the parasite's invasion efficiency to invade through the neuraminidase resistant (sialic acid independent) and trypsin resistant pathway [16]. On the other hand, the PfRH2b knock out displayed a higher rate of invasion with chymotrypsin treated erythrocytes inferring that the loss of the gene was allowing the parasite to invade better through the chymotrypsin resistant pathway and further implying PfRH2b to be involved in a chymotrypsin sensitive pathway [16]. Thus, while no direct evidence for erythrocyte binding of PfRH2b was reported, the knock out study showed that PfRH2b is involved in erythrocyte invasion and interacts with a sialic acid independent, trypsin resistant and chymotrypsin sensitive Receptor Z [16].

The PfRH2a knock out parasite line did not display any difference in invasion compared to the wild type 3D7 clone, which clearly suggested that PfRH2b had a more dominant role in erythrocyte invasion than PfRH2a in 3D7. This was further confirmed in another knock out study that found a stronger selection pressure for the reconstitution of PfRH2b expression at the expense of PfRH2a [25]. Further, genetic insertion of the PfRH2b gene in the D10 clone that endogenously lacks the PfRH2b gene but expresses PfRH2a significantly increased the efficiency of the transfected parasite line to invade neuraminidase and trypsin treated erythrocytes and simultaneously reduced its efficiency to invade chymotrypsin treated erythrocytes [17]. These results further substantiated the dominant role of PfRH2b over PfRH2a and confirmed its role in a sialic acid independent, trypsin resistant, chymotrypsin sensitive invasion pathway. However, a role of PfRH2a was demonstrated in the sialic acid independent pathway as knockouts of PfRH2a in the W2mef/Nm switched parasites reduced their efficiency to invade through the sialic acid independent pathway [16]. Thus, inspite of the high identity between PfRH2a and PfRH2b and the fact that our antibodies raised against a common region cannot differentiate between the two homologues, it is highly probable that our results reflect the functional activity of the dominant PfRH2b homologue as PfRH2b has a more dominant role in erythrocyte invasion than PfRH2a. Most importantly, we have demonstrated for the first time that the processed form of PfRH2a/b possesses erythrocyte binding activity and is involved in sialic acid independent invasion. Previously published PfRH2 antibodies were raised against regions near the C-terminal and not against the N-terminal region that exhibits erythrocyte binding activity [10], [16]. This is probably a major reason as to why previous studies have failed to detect erythrocyte binding of PfRH2 as the appropriate antibodies that could detect the processed N-terminal PfRH2 fragments were not used for erythrocyte binding assays.

Another significant finding of our work is the elucidation of the erythrocyte binding domain of PfRH2a/b. We have mapped the domain to a 40 kDa region (rPfRH2_40_, amino acids 495–860) that binds erythrocytes with the same specificity as that of the native 220 kDa PfRH2a/b protein and is consistent with the role of PfRH2a/b in the sialic acid independent, trypsin resistant and chymotrypsin sensitive pathway. We also found that antibodies against PfRH2a/b blocked erythrocyte invasion. Since, PfRH2b is a major parasite ligand mediating sialic acid independent invasion, we tested the ability of PfRH2a/b antibodies to block invasion of neuraminidase treated erythrocytes and observed that invasion blocking with neuraminidase treated erythrocytes is higher than with untreated normal erythrocytes. The reason for this higher invasion blocking efficiency is that with normal untreated erythrocytes, the parasites have a number of ligand-receptor interactions at its disposal to mediate successful invasion. These include both sialic acid dependent and independent parasite molecules. With neuraminidase treatment, the parasite is restricted to use only sialic acid independent pathways while other sialic acid dependent pathways are unavailable due to the removal of sialic acid moieties on the erythrocyte surface. Therefore, the PfRH2a/b antibodies blocked invasion of neuraminidase treated erythrocytes more efficiently than that of normal untreated erythrocytes. To prove that this inhibition was primarily due to antibodies against PfRH2, we were able to demonstrate that antibody inhibition could be neutralized by adding recombinant protein in the invasion assay well. This was further confirmed in invasion assays with the Dd2 parasite clone that invades erythrocytes only through sialic acid dependent pathways. Since PfRH2 is involved in sialic acid independent invasion, it would not be expected to block invasion by Dd2 parasites which do not use sialic acid independent pathways. Our invasion assay results were consistent with this prediction as anti-rPfRH2_40_ antibodies did not block erythrocyte invasion by the Dd2 clone.

Recently published data has shown that the N-terminal region of PfRH2 is more polymorphic than the rest of the protein and is a target of protective immunity against *P. falciparum* malaria [40]. An analysis of PfRH2 sequences among 33 parasite lines showed that the region (amino acids 212–745) was most polymorphic and suggested that it was under diversifying selection due to immune pressure [40]. The study found human antibodies against this N-terminal region showed the strongest association with protection from high density parasitemia. The polymorphic N-terminal region of PfRH2 has a substantial overlap with the rPfRH2_40_ region (amino acids 495–860) that we have demonstrated to be the erythrocyte binding domain of PfRH2a/b. It is evident that protein domains that bind with the host erythrocyte would be exposed to the immune system and thus be under immune pressure. Thus, this recent data further substantiates our finding of the erythrocyte binding domain of PfRH2.

The identification of the erythrocyte binding regions of invasion proteins is important as it helps in demarcating the critical functional domains of such large molecular weight proteins that could be further developed as sub-unit vaccines. We have not only demonstrated for the first time that PfRH2a/b like its other paralogues of the PfRH family is an erythrocyte binding protein, our report has also identified the functional domain of the PfRH2 protein that is responsible for mediating erythrocyte binding. We have expressed a region of PfRH2a/b that was homologous to the erythrocyte binding regions of PfRH1 and PfRH4. However, the exact boundaries of the receptor binding domain of PfRH2a/b remains to be further mapped. It is still possible that other regions of PfRH2a/b are also involved in erythrocyte binding and may come together in the three-dimensional structure to fully constitute the erythrocyte binding domain. The three dimensional structure of PfRH proteins has yet not been reported. As our understanding of the functional domains of the PfRH proteins has improved, the next challenge is the complete elucidation of the structure of the PfRH proteins. Thus, further research would be needed to completely dissect the structural and functional role of the PfRH proteins in merozoite invasion.

## Supporting Information

Materials and Methods S1 The Methodology used to study the localization of PfRH2a/b by immunofluorescence confocal microscopy is described.
(DOC)

Figure S1 **Alignment of the PfRH2b protein sequence with the amino acid sequence of PfRH1.** Pairwise clustal alignment of the PfRH2b (Genbank number AAN39447) protein sequence with PfRH1 (Genbank number AAQ10315). The 366 amino acid sequence of PfRH2a/b selected for recombinant expression (Ser 495 to Ile 860) is highlighted in the boxes. “*” identical residues; “**:**” conserved substitutions; “**.**” semi-conserved substitutions.
(TIF)

Figure S2 **Alignment of the PfRH2b protein sequence with the amino acid sequence of PfRH4.** Pairwise clustal alignment of the PfRH2b (Genbank number AAN39447) protein sequence with PfRH4 (Genbank number AAM47174). The 366 amino acid sequence of PfRH2a/b selected for recombinant expression (Ser 495 to Ile 860) is highlighted in the boxes. “*” identical residues; “**:**” conserved substitutions; “**.**” semi-conserved substitutions.
(TIF)

Figure S3 **Immunogenicity of the recombinant rPfRH2_40_ protein.** (A) The titers of antibodies raised against rPfRH2_40_ in five mice were measured in standardized ELISA. Three control mice immunized with adjuvant alone were also analyzed. Titers in the three control mice at a dilution of 1:1000 were extremely low and similar to the titers of the pre-immune sera from the five immunized mice. (B) Titers of anti-PfRH2_40_ antibodies were measured in rabbit sera. High titer antibodies (end point observed at dilution of 1:320,000 in mice and 1:640,000 in rabbits) against the recombinant rPfRH2_40_ protein were detected.
(TIF)

Figure S4 **SDS-PAGE of metal affinity chromatography purified proteins raised against different regions in the ectodomain of PfRH2a/b.** (A) rRH2-Pro1 (amino acids 76-494) and (B) rRH2-Pro4 (amino acids 1599-2059). The partially purified proteins were eluted from acrylamide and immunized in mice.
(TIF)

Figure S5 **Localization of PfRH2a/b by immunofluorescence confocal microscopy.** (A) 3D7 schizonts were dual labeled with anti-rPfRH2_40_ mice sera and anti-clag3.1 rabbit sera. Mature schizonts immunolabeled with anti-rPfRH2_40_ were stained with Alexa 488 linked anti-mouse IgG secondary antibody (green). Schizonts labeled with anti-clag3.1 rabbit sera were stained with Alexa 594 linked anti-rabbit IgG secondary antibody (red). (B) 3D7 mature schizonts were dual labeled with anti-rPfRH2_40_ mouse sera and anti-EBA175 rabbit sera. Schizonts labeled with anti-EBA-175 antibodies were stained with Alexa 594 linked anti-rabbit IgG secondary antibody (red). PfRH2a/b co-localizes with the known rhoptry marker protein, clag3.1 and not with the microneme marker protein, EBA-175.
(TIF)

Figure S6 (**A**) Full length native PfRH2a/b and its processed forms were detected in 3D7 parasite extracts by using a higher concentration of anti-rPfRH2_40_ sera. (B) Binding of the native PfRH2a/b protein in 3D7 culture supernatants incubated with untreated (U) erythrocytes, different enzyme-treated erythrocytes (Nm: neuraminidase-treated; T: trypsin-treated; C: chymotrypsin-treated). The processed 220 kDa and 80 kDa PfRH2a/b parasite proteins were detected in the eluate fractions by immunoblotting using antibodies against the rRH2-Pro1 region.
(TIF)
