# Supplementary material for: Plasmodium falciparum Reticulocyte Binding-Like Homologue Protein 2 (PfRH2) Is a Key Adhesive Molecule Involved in Erythrocyte Invasion
Source: PLoS One. 2011 Feb 28;6(2):e17102. doi: 10.1371/journal.pone.0017102 (PMC3046117; doi:10.1371/journal.pone.0017102)
Supplement: Materials and Methods S1 — The Methodology used to study the localization of PfRH2a/b by immunofluorescence confocal microscopy is described. (DOC) [file pone.0017102.s001.doc]

**Supplementary Text**

**Materials and Methods**

**Localization of PfRH2a/b by immunofluorescence confocal microscopy**

PfRH2a/b was localized in *P. falciparum* late stage schizonts by a confocal immunofluorescence assay (IFA) as described earlier [1]. Briefly, schizont-enriched parasites were smeared on slides and stored at -80^o^C. Slides were thawed, methanol-fixed and incubated with mouse sera raised against rPfRH2_40_ at room temperature for 1 hour followed by Alexa-fluor dye conjugated secondary antibodies (Invitrogen, Carlsbad, CA) at room temperature for 1 hour. Co-localization was performed using antibodies against specific organelle marker proteins such as EBA-175 for micronemes and Clag3.1 for rhoptries. Differential interference contrast (DIC) and fluorescence images were collected using a Nikon Model A1 confocal microscope (Nikon, Japan).

**References:**

1. [Singh S](http://www.ncbi.nlm.nih.gov/pubmed?term="Singh S"%5BAuthor%5D), [Alam MM](http://www.ncbi.nlm.nih.gov/pubmed?term="Alam MM"%5BAuthor%5D), [Pal-Bhowmick I](http://www.ncbi.nlm.nih.gov/pubmed?term="Pal-Bhowmick I"%5BAuthor%5D), [Brzostowski JA](http://www.ncbi.nlm.nih.gov/pubmed?term="Brzostowski JA"%5BAuthor%5D), [Chitnis CE](http://www.ncbi.nlm.nih.gov/pubmed?term="Chitnis CE"%5BAuthor%5D) (2010) Distinct external signals trigger sequential release of apical organelles during erythrocyte invasion by malaria parasites. PLOS Pathog 6(2):e1000746.
