# Supplementary figures and images for: Plasmodium falciparum Reticulocyte Binding-Like Homologue Protein 2 (PfRH2) Is a Key Adhesive Molecule Involved in Erythrocyte Invasion
Source: PLoS One. 2011 Feb 28;6(2):e17102. doi: 10.1371/journal.pone.0017102 (PMC3046117; doi:10.1371/journal.pone.0017102)

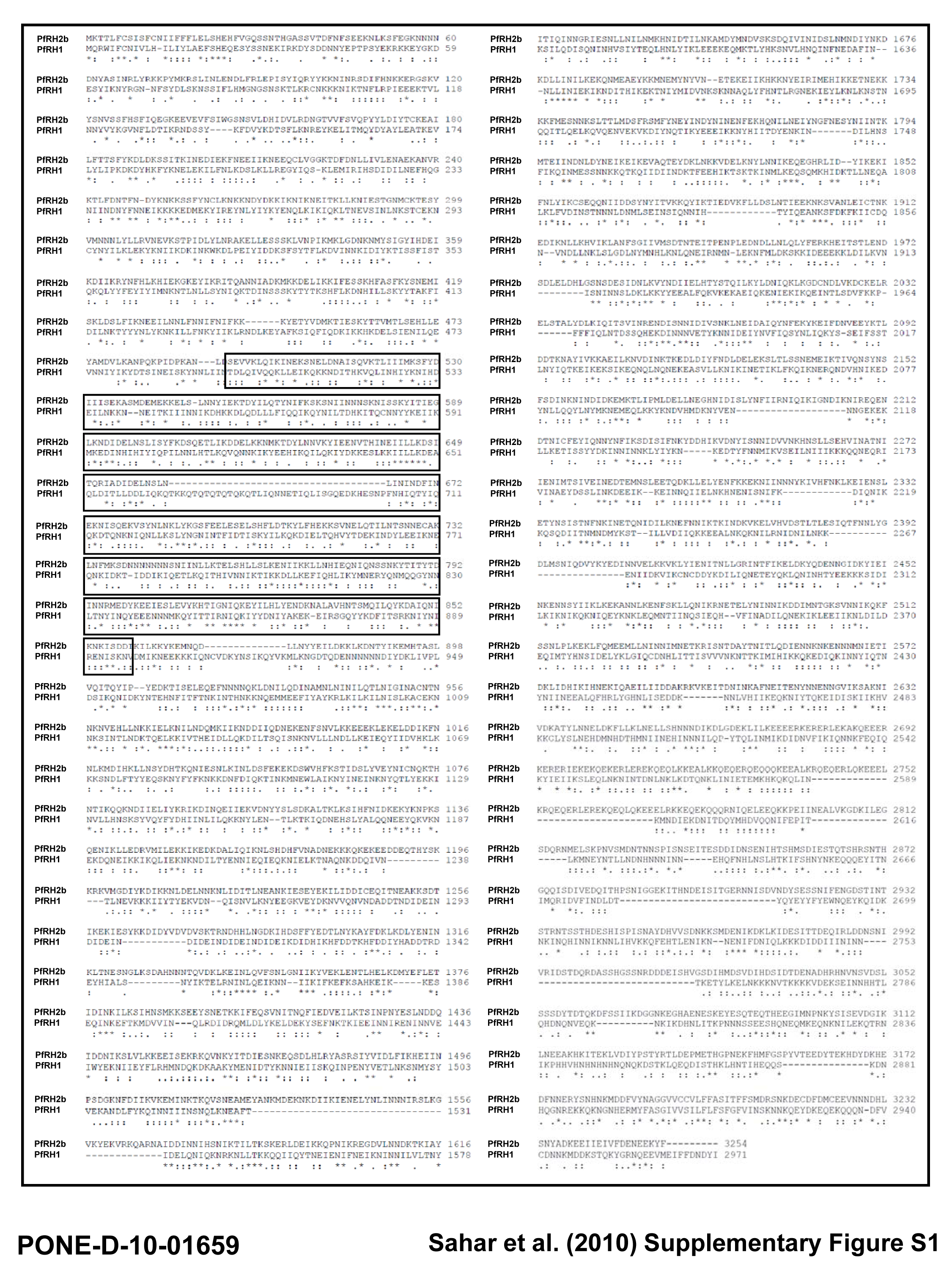

Supplement: Figure S1 — Alignment of the PfRH2b protein sequence with the amino acid sequence of PfRH1. Pairwise clustal alignment of the PfRH2b (Genbank number AAN39447) protein sequence with PfRH1 (Genbank number AAQ10315). The 366 amino acid sequence of PfRH2a/b selected for recombinant expression (Ser 495 to Ile 860) is highlighted in the boxes. “*” identical residues; “:” conserved substitutions; “.” semi-conserved substitutions. (TIF) [file pone.0017102.s002.tif]

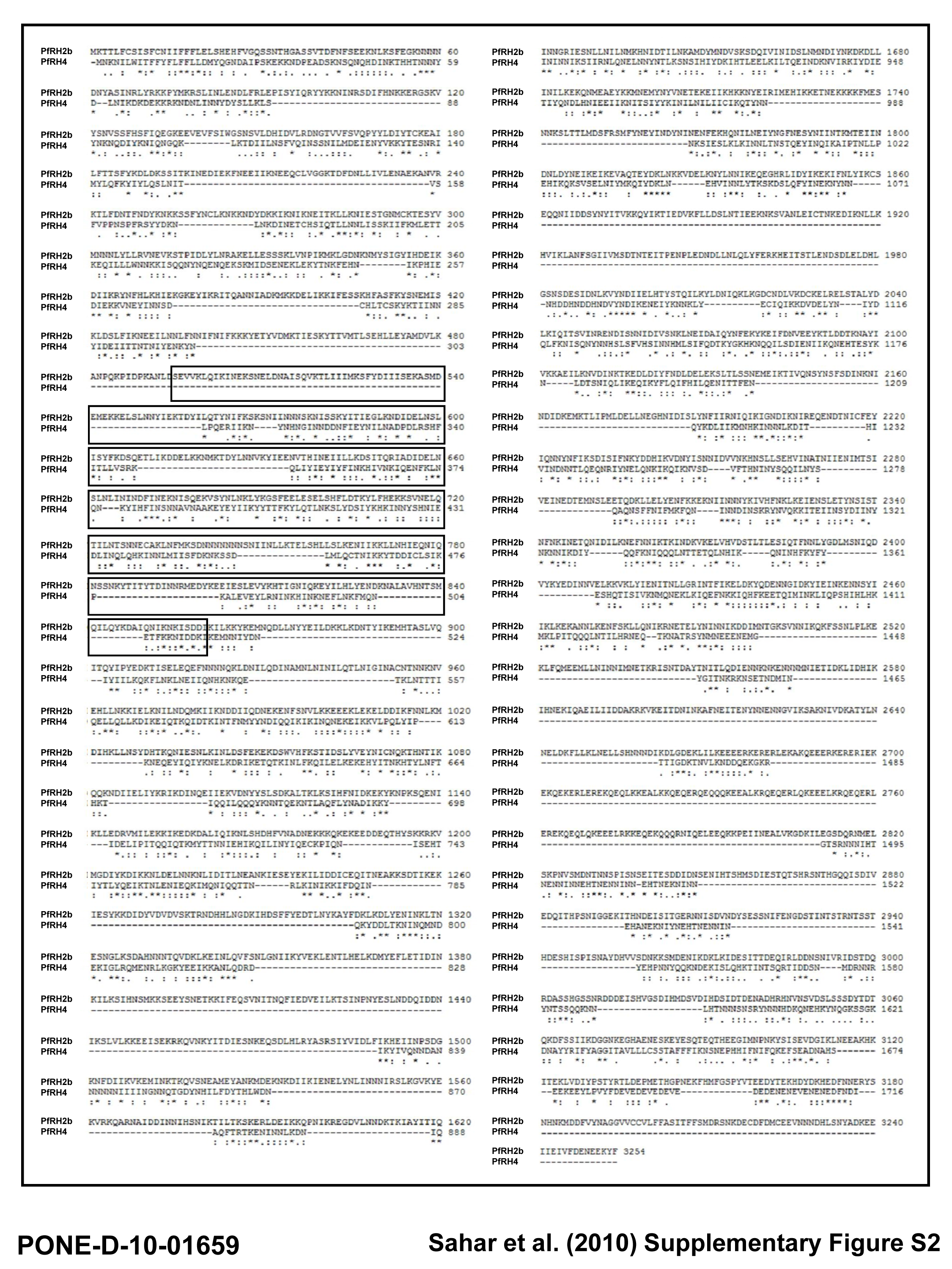

Supplement: Figure S2 — Alignment of the PfRH2b protein sequence with the amino acid sequence of PfRH4. Pairwise clustal alignment of the PfRH2b (Genbank number AAN39447) protein sequence with PfRH4 (Genbank number AAM47174). The 366 amino acid sequence of PfRH2a/b selected for recombinant expression (Ser 495 to Ile 860) is highlighted in the boxes. “*” identical residues; “:” conserved substitutions; “.” semi-conserved substitutions. (TIF) [file pone.0017102.s003.tif]

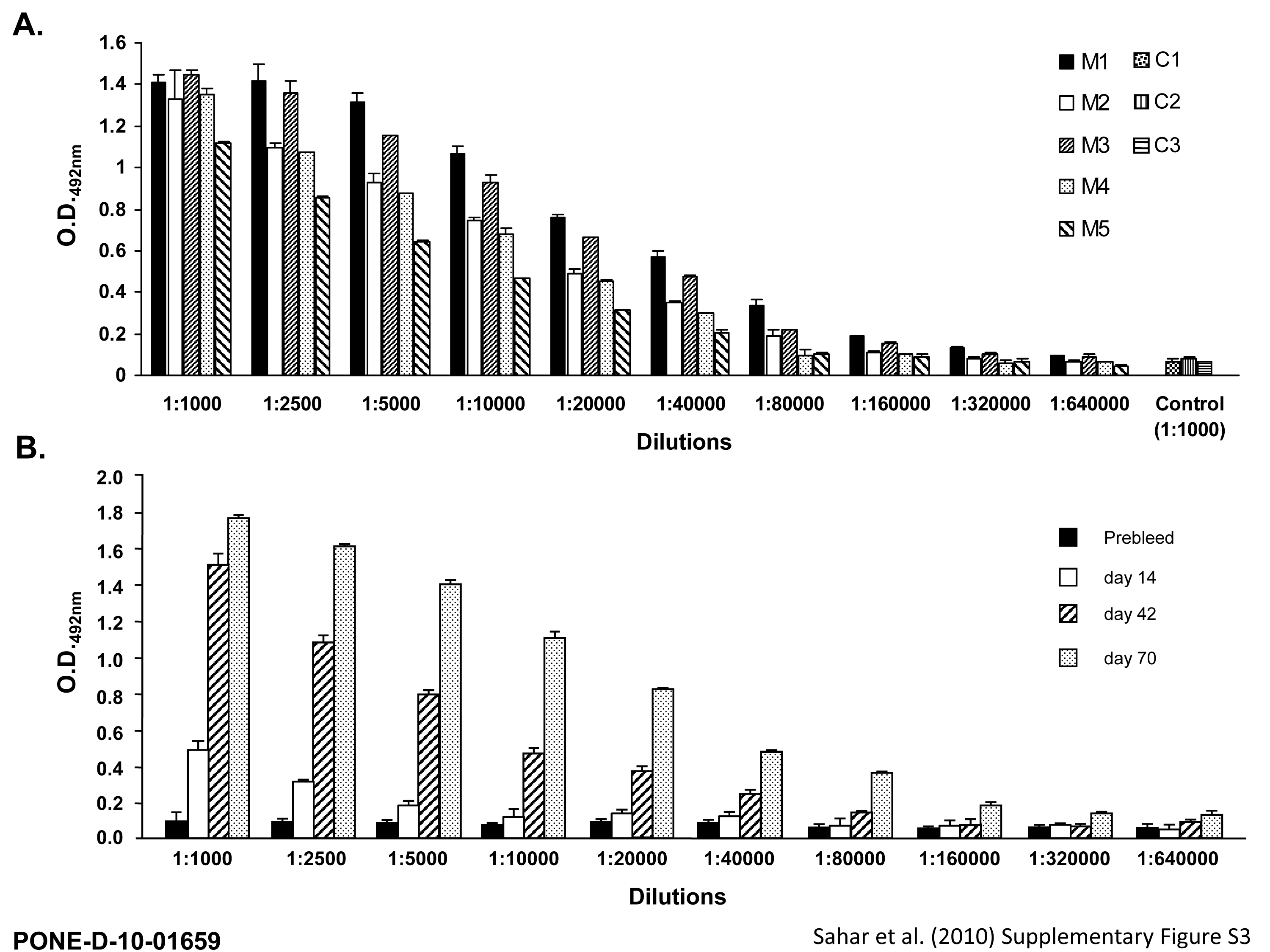

Supplement: Figure S3 — Immunogenicity of the recombinant rPfRH240 protein. (A) The titers of antibodies raised against rPfRH240 in five mice were measured in standardized ELISA. Three control mice immunized with adjuvant alone were also analyzed. Titers in the three control mice at a dilution of 1:1000 were extremely low and similar to the titers of the pre-immune sera from the five immunized mice. (B) Titers of anti-PfRH240 antibodies were measured in rabbit sera. High titer antibodies (end point observed at dilution of 1:320,000 in mice and 1:640,000 in rabbits) against the recombinant rPfRH240 protein were detected. (TIF) [file pone.0017102.s004.tif]

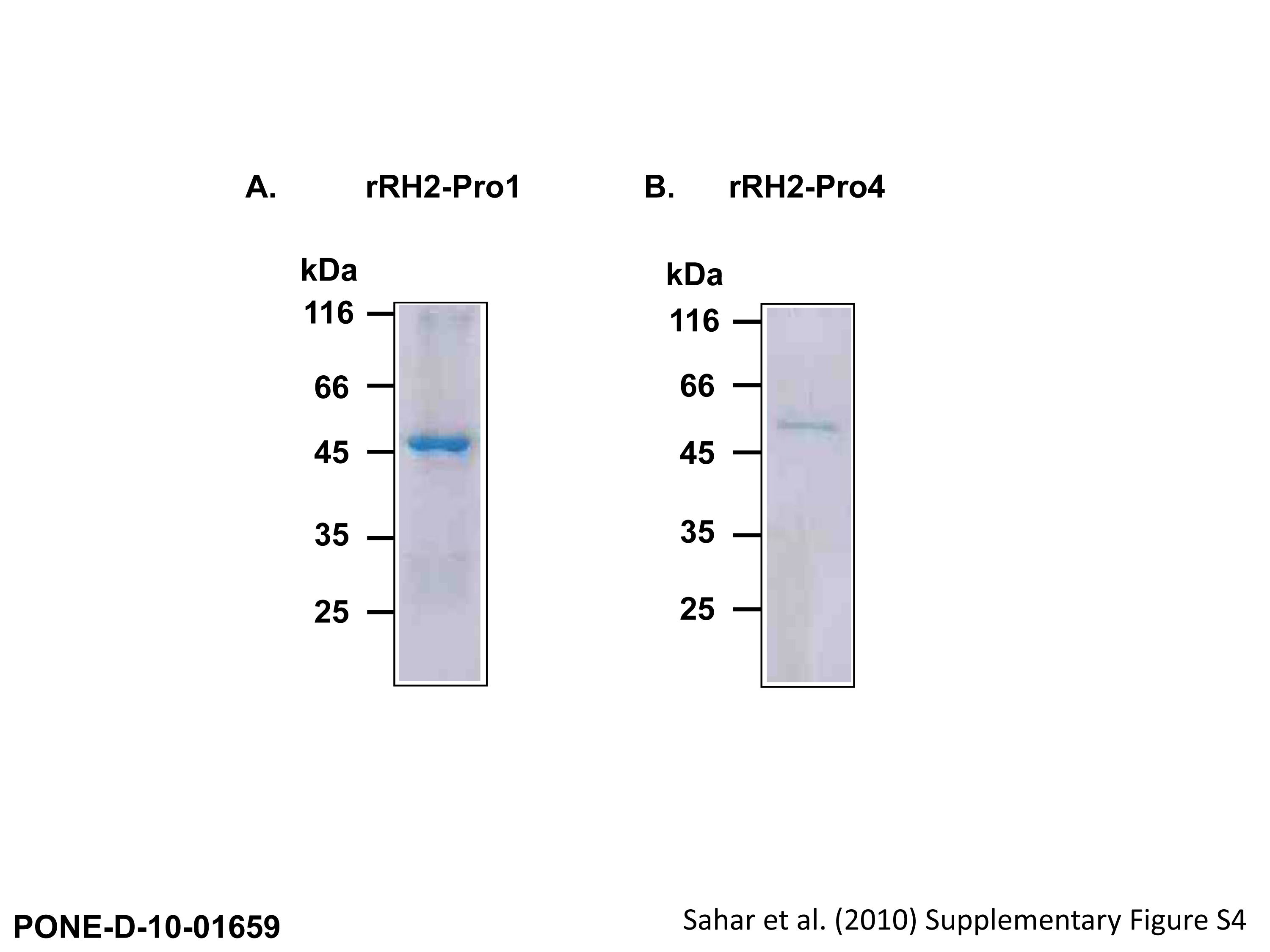

Supplement: Figure S4 — SDS-PAGE of metal affinity chromatography purified proteins raised against different regions in the ectodomain of PfRH2a/b. (A) rRH2-Pro1 (amino acids 76-494) and (B) rRH2-Pro4 (amino acids 1599-2059). The partially purified proteins were eluted from acrylamide and immunized in mice. (TIF) [file pone.0017102.s005.tif]

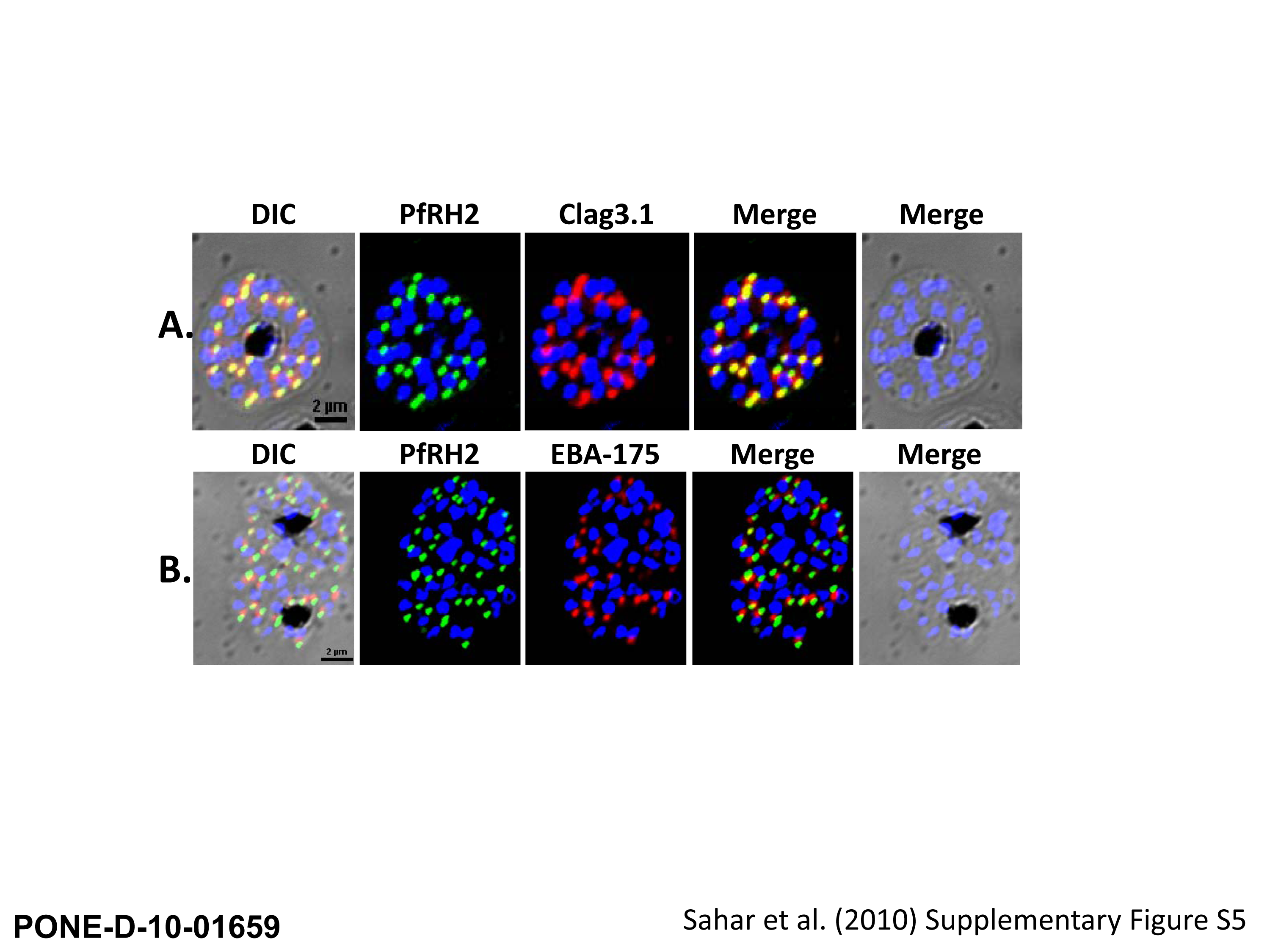

Supplement: Figure S5 — Localization of PfRH2a/b by immunofluorescence confocal microscopy. (A) 3D7 schizonts were dual labeled with anti-rPfRH240 mice sera and anti-clag3.1 rabbit sera. Mature schizonts immunolabeled with anti-rPfRH240 were stained with Alexa 488 linked anti-mouse IgG secondary antibody (green). Schizonts labeled with anti-clag3.1 rabbit sera were stained with Alexa 594 linked anti-rabbit IgG secondary antibody (red). (B) 3D7 mature schizonts were dual labeled with anti-rPfRH240 mouse sera and anti-EBA175 rabbit sera. Schizonts labeled with anti-EBA-175 antibodies were stained with Alexa 594 linked anti-rabbit IgG secondary antibody (red). PfRH2a/b co-localizes with the known rhoptry marker protein, clag3.1 and not with the microneme marker protein, EBA-175. (TIF) [file pone.0017102.s006.tif]

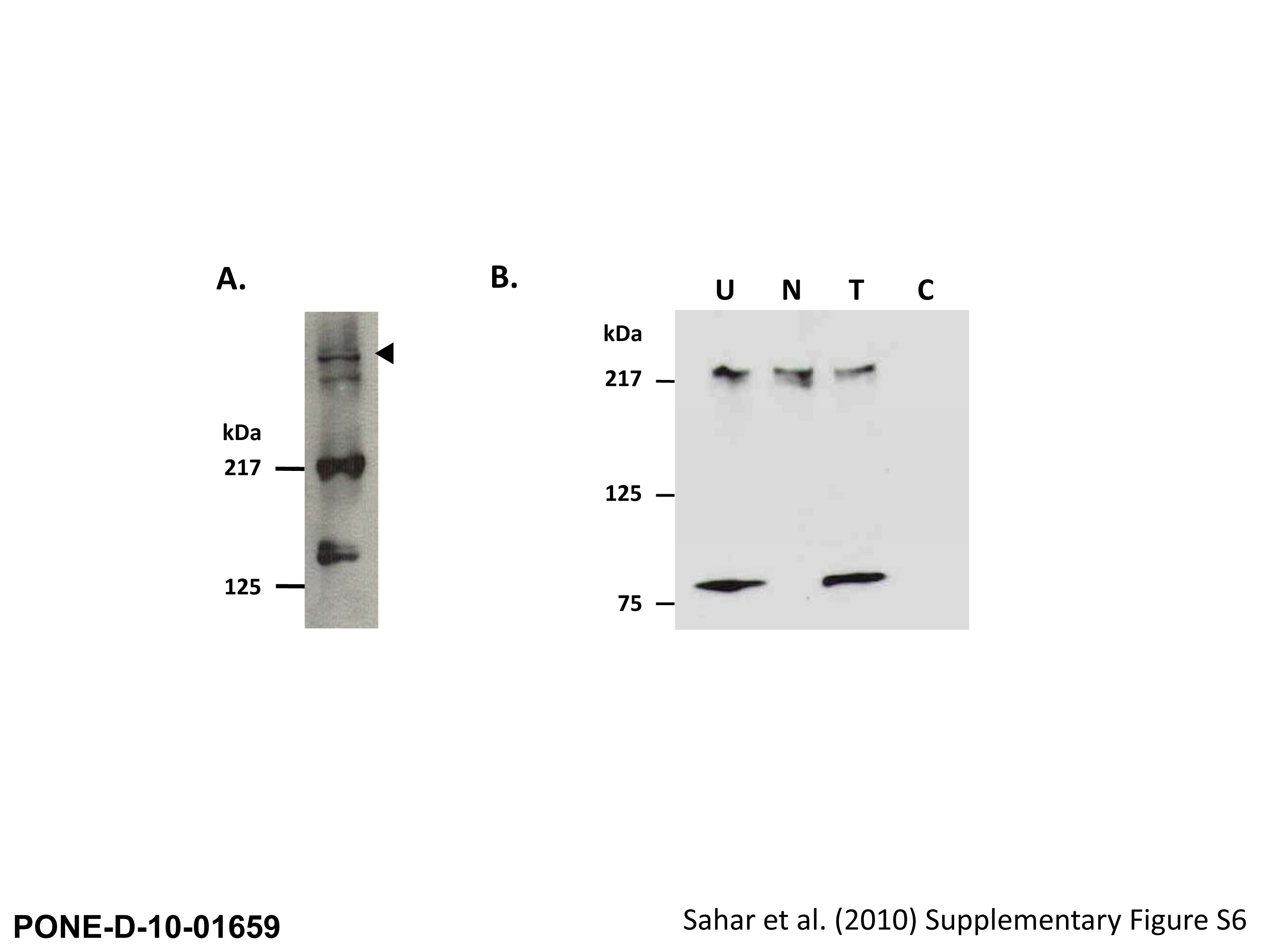

Supplement: Figure S6 — (A) Full length native PfRH2a/b and its processed forms were detected in 3D7 parasite extracts by using a higher concentration of anti-rPfRH240 sera. (B) Binding of the native PfRH2a/b protein in 3D7 culture supernatants incubated with untreated (U) erythrocytes, different enzyme-treated erythrocytes (Nm: neuraminidase-treated; T: trypsin-treated; C: chymotrypsin-treated). The processed 220 kDa and 80 kDa PfRH2a/b parasite proteins were detected in the eluate fractions by immunoblotting using antibodies against the rRH2-Pro1 region. (TIF) [file pone.0017102.s007.tif]
